# Determinants of Successful eHealth Coaching for Consumer Lifestyle Changes: Qualitative Interview Study Among Health Care Professionals

**DOI:** 10.2196/jmir.9791

**Published:** 2018-07-05

**Authors:** Carl Joakim Brandt, Gabrielle Isidora Søgaard, Jane Clemensen, Jens Søndergaard, Jesper Bo Nielsen

**Affiliations:** ^1^ Research Unit of General Practice Department of Public Health University of Southern Denmark Odense C Denmark; ^2^ Centre for Innovative Medical Technology University of Southern Denmark Odense Denmark; ^3^ Hans Christian Andersen’s Children’s Hospital Odense University Hospital Odense Denmark

**Keywords:** behavior change, eHealth coaching, empathy, lifestyle, healthy lifestyle, mHealth, mobile health units, primary health care, primary care, public health, telemedicine

## Abstract

**Background:**

Success with lifestyle change, such as weight loss, tobacco cessation, and increased activity level, using electronic health (eHealth) has been demonstrated in numerous studies short term. However, evidence on how to maintain the effect long-term has not been fully explored, even though there is a pressing need for long-term solutions. Recent studies indicate that weight loss can be achieved and maintained over 12 and 20 months in a primary care setting using a collaborative eHealth tool. The effect of collaborative eHealth in promoting lifestyle changes depends on competent and skilled dieticians, nurses, physiotherapists, and occupational therapists acting as eHealth coaches. How such health care professionals perceive delivering asynchronous eHealth coaching and which determinants they find to be essential to achieving successful long-term lifestyle coaching have only been briefly explored and deserve further exploration.

**Objective:**

The aim of this study is to analyze how health care professionals perceive eHealth coaching and to explore what influences successful long-term lifestyle change for patients undergoing hybrid eHealth coaching using a collaborative eHealth tool.

**Methods:**

A total of 10 health care professionals were recruited by purposive sampling. They were all women aged 36 to 65 years of age with a mean age of 48 years of age. A total of 8/10 (80%) had more than 15 years of experience in their field, and all had more than six months of experience providing eHealth lifestyle coaching using a combination of face-to-face meetings and asynchronous eHealth coaching. They worked in 5 municipalities in the Region of Southern Denmark. We performed individual, qualitative, semistructured, in-depth interviews in their workplace about their experiences with health coaching about lifestyle change, both for their patients and for themselves, and mainly how they perceived using a collaborative eHealth solution as a part of their work.

**Results:**

The health care professionals all found establishing and maintaining an empathic relationship essential and that asynchronous eHealth lifestyle coaching challenged this compared to face-to-face coaching. The primary reason was that unlike typical in-person encounters in health care, they did not receive immediate feedback from the patients. We identified four central themes relevant to the health care professionals in their asynchronous eHealth coaching: (1) establishing an empathic relationship, (2) reflection in asynchronous eHealth coaching, (3) identifying realistic goals based on personal barriers, and (4) staying connected in asynchronous coaching.

**Conclusions:**

Establishing and maintaining an empathic relationship is probably the most crucial factor for successful subsequent eHealth coaching. It was of paramount importance to get to know the patient first, and the asynchronous interaction aspect presented challenges because of the delay in response times (both ways). It also presented opportunities for reflection before answering. The health care professionals found they had to provide both relational communication and goal-oriented coaching when using eHealth solutions. Going forward, the quality of the health care professional–patient interaction will need attention if patients are to benefit from collaborative eHealth coaching fully.

## Introduction

Successful electronic health (eHealth) lifestyle coaching to increase exercise, improve diet, and reduce tobacco and alcohol use has been demonstrated in numerous studies [1].However, maintaining the effect over extended periods of time has had more variable results [2]. New studies have demonstrated remission from a diabetic state for almost half of a patient population solely by increased activity, diet, and weight loss in both primary and secondary care settings [3]. A recent study showed that 96% of a representative sample of 1004 Danes between 40-60 years of age preferred lifestyle change to medication [4] even though few general practitioners recognize this [5].

Many studies show that empathy by the health care professional (HCP) providing the lifestyle coaching is of paramount importance for in-person coaching [6,7]. Previously, we reported on a collaborative eHealth solution that resulted in long-term behavioral change where weight loss of 7.0 kg over 20 months was achieved using eHealth coaching in a general practice setting [8]. The same findings were observed in a municipality setting with diabetic men, where patients stated that an initial in-person meeting with the dietician seemed critical for their future Web-based interaction [9]. Other studies suggest that HCPs enjoy in-person meetings more than eHealth coaching [10]. Despite the success of these smaller-scale studies, there is a need to clarify various aspects of eHealth coaching and factors influencing successful long-term lifestyle change [11]. Use of eHealth is viewed positively by general practitioners (GPs), who use motivational interviewing in their practices and eHealth for their health [5].

The importance of the HCPs’ support of patients with lifestyle challenges and how the HCPs perceive the use of eHealth has not yet been explored [12]. Hence, we aimed to identify factors essential to HCPs assisting patients undergoing lifestyle changes using eHealth. Of particular focus was how the HCPs viewed their eHealth coaching, what motivated them, and which factors in their eHealth coaching were most important for supporting their patients and guide them through the challenges faced on the way towards a healthier lifestyle.

## Methods

### Context

Denmark and the Danish health care sector have 3 political and administrative levels: the national state, 5 geographically defined regions, and 98 municipalities. Municipalities have on average approximately 57,000 inhabitants. They are local administrative bodies and deliver public health care, disease prevention, and rehabilitation at the local level, outside of hospitals [13].

### Design

This qualitative study was based on in-depth and semistructured individual interviews with 10 Danish HCPs who provide eHealth coaching in health care centers in 5 municipalities in the Region of Southern Denmark. HCPs in a municipality health care center can have different health care education backgrounds including dieticians, physiotherapists, nurses, and occupational therapists.

### Sampling

Sampling was conducted among 12 female HCPs providing eHealth coaching, who had coached more than 30 patients for more than 3 months, and individuals were recruited by email or phone. In total, 11 HCPs were invited, although 1 declined to participate due to a job change. Saturation was met after 7 interviews, but the remaining 3 interviews were conducted to confirm that no new themes or subthemes emerged [14]. The HCPs interviewed were all female, between 36-65 years of age, with a mean age of 48 years of age. A total of 8/10 (80%) had more than 15 years of experience in their field. All had experience providing hybrid eHealth lifestyle coaching using a combination of synchronous face-to-face meetings and asynchronous eHealth coaching through a collaborative eHealth tool. There were 10 female HCPs, including 5/10 (50%) clinical dieticians, 2/10 (20%) physiotherapists, 1/10 (10%) nurse, 1/10 (10%) occupational therapist, and 1/10 (10%) nurse assistant. Half 5/10 (50%) had taken specific postgraduate coaching courses in motivational interviewing, and 2/10 (20%) had other pedagogic educations. They had between 0.5-31 years of coaching experience. A total of 9/10 (90%) had other tasks, such as coaching or teaching patients in traditional face-to-face coaching or group sessions. They spent 4-16 hours per week on asynchronous eHealth coaching, and interacting with 20-140 current patients through the collaborative eHealth tool.

### Interview Procedure

An explorative approach was followed in order to explore the HCPs´ subjective experiences and interpretations of working with eHealth coaching, focusing on motivational factors for a successful long-term lifestyle change. Semistructured interviews were conducted with the participating HCPs, following a basic, loose interview guide with overall fields of interest and probing questions that permitted in-depth exploration of the HCPs´ views and perceptions (see Table 1). The question guidelines helped the researcher (CJB) to follow an iterative approach with room for exploration of emerging themes and perspectives that could be further explored in interviews with subsequent participants [15].

**Table 1:** Interview guide for semistructured interviews with health care professionals.

Fields of interest	Probing questions	
Experience with health coaching involving patients with lifestyle challenges in the municipality health center	Please tell me about good and bad experiences you have had with health coaching. Why do you think it played out that way?	
Their own lifestyle experiences	Have you ever taken the initiative to improve or change your lifestyle? Do you use experiences from your own life in your coaching? Why or why not?	
Experience with electronic health and digital coaching in relation to their own and patients’ health challenges	How much experience do you have with communicating with patients using digital tools? What works well and what does not work well in digital coaching?	

The interviews were carried out in the HCPs’ offices from May to June 2017 and took 45-75 minutes each. All interviews were performed by CJB, who has worked as a GP for more than ten years and with different eHealth solutions for more than fifteen years.

### Ethical Considerations

The ethics committee for the Region of Southern Denmark considered that the protocol could be approved and determined that the Medical Research Involving Human Subjects Act does not apply to this study [16]. All participants were informed of CJB’s role as a GP, and shareholder of Liva Healthcare A/S that delivered part of the software. It was emphasized that CJB would interview them as a researcher.

Before an interview was initiated, CJB briefly explained the nature of the research, answered any questions regarding the study, and described the study in layman’s terms. The participants were informed of their rights, and CJB explained that the interview data would be anonymized. Both the participant and CJB signed informed consent documents. Emails and phone numbers were obtained from the municipalities before the study commenced. Researcher CJB invited the participants, made arrangements for the interviews and handled all phone calls and email correspondence regarding this matter.

### Intervention

HCPs conducted eHealth coaching using the collaborative eHealth solution LIVA [17] in a hybrid manner, combining face-to-face meetings with eHealth coaching. LIVA is a refinement of the former eHealth solutions Slankedoktor.dk [8] and mydietician.org.uk [9], which were used and described in detail in 2 previously reported studies [8,9]. The 5 participating municipalities have offered this eHealth tool to patients for 6-12 months and have each included 100-400 patients. Patients using the eHealth solution report on individual goals in real-time including activity, diet, sleep, pain, and compliance with personal goals or other goals agreed on with the HCP, via iOS, Android or web. HCPs used a Web-based “backend” interface that served as a control panel, cockpit, and library. eHealth coaching is conducted asynchronously via short message service text messaging, or video messaging weekly, biweekly, monthly, or in a way the HCP decided was most appropriate to meet the patients’ needs. In Multimedia Appendix 1, the eHealth solution LIVA is presented with detail inspired by the Template for Intervention Description and the Replication checklist [18], with information on the specific behavioral change techniques from the Coventry, Aberdeen and London-Refined taxonomy [19].

### Analyses

The 10 interviews were digitally recorded and transcribed verbatim. Analyses were performed by the researchers (CJB, GIS, JC, JBN, and JS) using thematic analysis. An explorative approach of systematic text condensation was applied [20,21]. The analysis process began with all researchers reading through the transcripts. They gained their impressions of what they viewed as relevant and exciting themes and then met several times to discuss their different views and agree upon a “codebook” of categorized ideas and topics within specific themes and subthemes relevant for the set objectives. The researchers CJB and GIS then started the a priori coding of each transcript in the software program NVivo 11 Pro for Windows [22]. This was performed using a node structure that reflected identified themes and subthemes and allowed for expansion and reduction along the way. To make sure that the researchers coded, sorted and categorized the data in the same way—by identifying similar expressions, patterns, and sequences in the transcripts—the coding comparison function in NVivo 11 Pro was used on the first 3 interviews, and then coding was aligned where necessary. The data from each of the identified themes were then condensed and summarized into generalized descriptions and concepts. In the analysis process, the researchers related the extracted information to the full transcripts to make sure they preserved the original context. The identified themes were compared between the different researchers several times throughout the process. In the end, these descriptive themes were put into analytical themes according to the thematic synthesis approach [23]. Finally, the quotes that best illustrated each theme and its related subthemes were selected and translated from Danish to English. The researchers CJB and GIS initiated the translation process by comparing their translations, agreeing on wording and meaning in the sentences, and then comparing them a second time to the Danish quotes. The remaining authors then reviewed all quotes in Danish and English, and changes were made if all parties agreed. In the text, interview quotes are followed by a unique participant identifier, ranging from Health Care Professional 1 to Health Care Professional 10.

## Results

### Themes and Subthemes

We identified 4 central themes with many subthemes concerning the HCPs’ perceptions of conducting eHealth coaching (see Textbox 1 for an overview of these themes and their related subthemes):

1. Establishing an empathic relationship
2. Reflection in asynchronous eHealth coaching
3. Identifying realistic goals based on personal barriers
4. Staying connected in asynchronous coaching

### Establishing an Empathic Relationship

All HCPs found it challenging to provide proper eHealth coaching because it was not possible to get face-to-face feedback. Their typical tools to elicit feedback, such as mirroring body language or prompting patients to continue a line of thought by repeating the last word in a sentence, were not applicable since they were separated from their patients in time and space.

#### Combining Synchronous Face-to-face Coaching with Asynchronous eHealth Coaching

All HCPs found it essential to build an empathic relationship with room for reflection in face-to-face meetings before asynchronous eHealth coaching.

In this relationship we have built up, (by meeting face-to-face initially), they will tell you more personal things—at least that is what I experience—more than they did earlier on with Slankedoktor (digital-coaching only). I have coached one that has admitted excess eating, one that has told me that her daughter is at a crisis center, and it was very natural for them to share this.

Health Care Professional 10

#### Use the Health Care Professional’s Own Story About Lifestyle Change

To get to know the patient better and connect with the patients, HCPs found it beneficial to tell their own stories about lifestyle change. A total of 9/10 (90%) HCPs found it essential to show the patients that they knew that lifestyle changes required hard work. There were 7/10 (70%) HCPs that used their own experiences when they explained to patients what was needed to achieve a specific outcome. They often also made an effort to explain that despite looking healthy and fit, they also experienced challenges on a daily basis in maintaining their good health.

It is important for me to tell them that I am no bikini model, and I have been 25 kg heavier than I am now, so this is to say I know the kind of problems that matter on a daily basis.

Health Care Professional 4

**Themes and subthemes for using a collaborative electronic health (eHealth) tool in combination with face-to-face consultations for health care professionals.**

**Theme 1: Establishing an empathic relationship**

- Combining synchronous face-to-face coaching with asynchronous eHealth coaching
- Use the health care professional’s own story of lifestyle change
- Appreciating the communication in asynchronous eHealth coaching
- Health care professional’s motivation

**Theme 2: Reflection in asynchronous** **eHealth** **coaching**

- Health care professional reflection
- Patient reflection
- Explore individual motivation

**Theme 3: Identifying** **realistic goals based on personal barriers**

- Recognize harmful patterns
- Operational goal setting
- Appreciate small steps

**Theme 4: Staying** **connected** **in asynchronous coaching**

- Personal comments
- Reading the patient
- Feedback stimulated by open questions

#### Appreciating Communication in Asynchronous eHealth Coaching

When conducting eHealth coaching after the initial face-to-face meeting, HCPs found it essential to send messages with positive expectations, not only for measurable outcomes but also for communication itself, which was viewed as critical for patients to stay connected.

The patients tell me things like “I am always happy when I read what you have sent to me…”; “I look forward to seeing what you have written…” and one said“I am always so excited to see if you have found something I have done right.”

Health Care Professional 7

When I do digital coaching…I recognize that it has been difficult for the patient, or praise when I can see they are doing good. It can be a few words I send off or a video greeting. I really like video, because they say to me “I can feel you, it is like you are sitting on my shoulder cheering!”

Health Care Professional 8

Half of the HCPs (5/10, 50%) experienced nonjudgmental communication in “neutral waters”. For example, greetings for the holiday seasons, resulted in many more responses in comparison to when they asked for performance data or sent out standard messages with health educational content.

Some of the patients I had not heard from, but then I wrote that I had to take a leave the next two weeks because I had broken my arm, then it was almost everyone who commented and wished me good health.

Health Care Professional 1

#### The Health Care Professional’s Motivation

A total of 8/10 (80%) HCPs explained that they were motivated by meeting with another person, establishing a relationship, and getting closer to an “understanding” and “feeling” of the person in front of them. Even though many patients today are accustomed to digital communication, all HCPs found that an initial face-to-face meeting before initiating digital coaching was necessary to establish a strong and compassionate relationship.

I think that I was the factor that made the difference, since he (the patient) knew that I was the person who was coaching him. He had met me in person and it made a difference that it was not just another app he could use for entering his data. Here, he actually got concrete answers to his questions.

Health Care Professional 9

The coaching could only work if the patient communicated, and losing feedback was therefore seen as a significant challenge for all the HCPs.

Well, it motivates me when I get some kind of feedback from the patients. Then I think it is fun and nice to spend time on it. Those who do not give very much can be less motivating, I think.

Health Care Professional 10

### Reflection in Asynchronous eHealth Coaching

The HCPs found that they could deliver advice with the use of very little time working asynchronously. A total of 9/10 (90%) HCPs said that they used only 5-10 minutes for each digital coaching session. In comparison, face-to-face coaching tends to be very time consuming for both the patient and the HCP (ie, 30-60 minutes). The lack of direct patient interaction in asynchronously coaching challenges the coach’s abilities to see the patient’s reaction to advice or questions directly. However, this opens for reflection for both the HCP and the patient. Individual motivation also needs to be explored in manners other than known from traditional motivational interviewing.

#### Health Care Professional Reflection

Due to the time difference between when patients enter data and when advice is given, the HCPs could think, reflect, and adjust their advice before sending it to the patient. The HCPs did not have to answer immediately when they saw data from the patient. Instead they could go for a walk or answer another question before they returned to give personalized advice regarding what they had observed.

So you can stop (your digital consultation) and reflect: “What is it she really needs?”…Then you can come back later and finish your consultation.

Health Care Professional 7

#### Patient Reflection

In the same manner, when advice was given, patients had time before responding, which could be seen as a chance to think, reflect, comment, or enter other data. There was 1/10 (10%) HCP who explained how she saw this as an advantage for the patient when difficult topics need to be dealt with:

One patient once told me: “You can write it in small pieces if it really hurts (eg, difficult to talk about), as opposed to when you meet at the doctor’s surgery, at the dietician or at the psychologist you need to finish, you must say everything in the consultation right away.You cannot take a break, think about it and reply…”

Health Care Professional 7

#### Explore Individual Motivation

All HCPs found it essential to find out what motivated the patient. To accomplish this, they found it very important to give the patient space to reflect and initiated the coaching by providing the patient time for goal setting and reflection. Learning could then come from the lived life.

This man had diabetes, and he knew all about it, but he lacked ownership, and he did not understand how to cope with it. So, after he began here in the municipality center and we found out what help he needed, he began exercising and measuring his blood sugar. So now he has lost 20 kg, and he sees how exercise and healthy eating affect his blood sugar. He is really motivated when he sees the immediate effect and it is thought-provoking that, actually, he has never really understood the effects of carbs on the blood sugar (until now).

Health Care Professional 5

### Identifying Realistic Goals Based on Personal Barriers

During the digital coaching sessions, 9/10 (90%) HCPs found it essential to getting to know the patient better to understand if they had destructive patterns and to identify realistic goals. This helped them to recognize patients’ progress even though the patients did not see it themselves.

#### Recognize Harmful Patterns

The HCPs were often occupied with helping patients break free from harmful patterns and actions.

So, what can be the reason a person chooses to say that: “I cannot do it because of this and that.” That is to say, what is the reason he only sees barriers, and is it a pattern he has had throughout life?

Health Care Professional 1

#### Operational Goal Setting

The collaborative eHealth tool supported specific goals set out by the patient. Helping patients to be concrete and operational in their goal setting was mentioned by 5/10 (50%) HCPs as a challenge. As an example, moving from the generic *“I want to live healthy”* to the specific *“I want to eat breakfast”* was of vital importance when patients monitored their daily performance; operational goal-setting was crucial to turning goals into measurable outcomes.

Sometimes they are just not precise enough. Some of them might want to “eat healthy”, but what is it exactly they want to change? They need to be more concrete and specific about their challenges. Is it snacking in between meals that needs to be changed? Or what is it?

Health Care Professional 5

#### Appreciate Small Steps

In coaching sessions, 6/10 (60%) HCPs found it was important to recognize small signs of progress that might not be noted by the patient.

The patient could say: “I have not done anything since we last spoke”. When you then look closer and see that they have done something, but just not reached the goals they had expected…So, you move focus to their successes.

Health Care Professional 7

### Staying Connected in Asynchronous Coaching

All HCPs found it quite challenging when the patient did not respond to the eHealth coaching: if they took a very long time to reply, did not register their activities on a regular basis, or did not respond to the advice given in the last coaching session. A total of 9/10 (90%) HCPs explained that the lack of feedback often paused the process and made the HCP wonder what was going on—a situation that was new to them and indicated a need to approach things differently from what they had been used to in face-to-face coaching sessions.

So yes, using a collaborative eHealth tool really requires patience, because it takes a long time to get the answers. So, it has also been a process that has stretched over a long period of time, where I have asked her (the patient) a question, and I have added some reflective notes to it. And then I have waited for her answer before I could go on with the process. So, it is a different form, but I actually think that it has worked, yes! But you need to learn to accept, especially in the beginning, that it takes a long time, and that it is okay.

Health Care Professional 3

The HCP’s had developed many strategies to stay connected through personal comments, reading the patient, and using open questions.

#### Personal Comments

The eHealth solution provided the opportunity for the HCP to reuse “standard advice”. The HCPs explained that more than 50% of the content provided as either written or video advice that was reused. There were 6/10 (60%) HCPs explained that they made an effort to craft a unique, personalized, nonjudgmental frame around the necessary standard advice.

the specific advice is about 80% reuse, but I do make some small adaptations.

Health Care Professional 2

The tone I answer in will be unique and tailored to the individual—articles and recommendations will be reused of course—but the frame around it will always be unique. And then there will be prefabricated elements which are the same for everybody because it cannot be said in any other way.

Health Care Professional 7

#### Reading the Patient

The eHealth solution also allowed both the HCP and the patient to go back to an earlier question or answer to clarify what had been communicated. The HCPs highlighted this as positive for the interaction and very useful in situations where the HCP was uncertain about whether the patient shared the HCP’s view of the content of the communication.

I start looking for their registrations to see if there is something positive to comment on. Then I often start there…when I have said something or done something in the last communication we have had, then in the next message I send out to the person I kind of remind myself that I have to ask them whether they found it useful or not, just to give them the chance to say: “Well…I think it was a bit far out!”

Health Care Professional 6

#### Feedback Stimulated by Open Questions

Feedback had to be stimulated in different ways than what HCPs were used to in face-to-face interactions. Most HCPs tried to encourage more frequent feedback by sending very open but positive questions and remarks.

I asked the open question: “What do you eat and when do you eat?” and then I let her tell me herself. Then I asked her: “I can see you mention something you call junk and unhealthy stuff.” Then she replies: “I do not really eat much of that, but I eat large portions. My stomach has been accustomed to that.”

Health Care Professional 1

I always praise them for the work: “well done” and make an effort to write my reply so that it mirrors the themes they have mentioned as important to them.

Health Care Professional 10

## Discussion

Establishing and maintaining an empathic relationship with the patients was the single most crucial factor for the HCPs when they performed asynchronous eHealth coaching. This is in line with findings suggesting that lack of an empathic relationship with the patient can be toxic when providing motivational coaching [6].

### Establishing an Empathic Relationship

Empathy is an independent contributor to the benefit of behavioral interventions [24]. However, empathy is difficult to maintain in eHealth due to the need for mutual confirmation that happens through signs and signals when coaches interact with someone from their own “tribe” [25]. This tribal verbal and nonverbal language is fundamentally challenged by communicating digitally. The Internet provides access to an information overload that can be difficult to interpret for patients with low health literacy [26]. Our study suggests that if a trustworthy relationship is established and maintained, HCPs using hybrid eHealth coaching methods could be very useful for patients with low health literacy.

A systematic review of previous systematic reviews of studies using Web-based weight loss interventions revealed conflicting results for effects when comparing Web-based interventions with hybrid interventions [2]. Earlier studies on the consultation process revealed that health professionals only have access to a patient’s reflections on difficult, personal and relevant subjects about their health if the HCP manages to establish an empathic relationship [27]. Using hybrid, complex interventions, meeting patients both in-person (ie, synchronous) to strengthen relations and through asynchronous eHealth might improve health care through more effective and efficient interpersonal communication, even though long-term studies beyond 24 months are still missing [1,9]. A pilot randomized controlled trial study on feasibility and acceptability of a prior, Web-based version of the asynchronous eHealth tool used in this study for men with type 2 diabetes revealed that eHealth can facilitate relevant in-time feedback and profound reflections from the patients. Moreover, the effect of the intervention seems better when an empathic relationship between the patient and the HCP is established before the Web-based intervention is initiated [9]. HCPs used stories about their own health challenges to find common ground with the patients. For decades, health professionals have been warned against using their own personal health experiences in patient treatment. Moving into the 21^st^century, this notion might be challenged by successes with collaborative hybrid eHealth solutions, where empathy between patients and HCPs is a pivotal factor in securing long-term success [24]. Empathy may prove difficult to establish without HCPs using personal stories. We found that HCPs, who repeatedly appreciated communication through positive reinforcement of the asynchronous communication and not only the measurements registered seemed most successful in engaging patients and maintaining an empathic relation. Appreciating patient communication through text and video actively might be the HCPs way to express “reflective listening” digitally, known in the behavioral change theory [6]. The success of traditional motivational coaching depends on the empathy of the HCP [6]. Our study shows that empathy by the HCP can be challenged in asynchronous eHealth coaching. It, therefore, seems to be of paramount importance also to focus on what motivates the HCP and how their self-empathy can be nourished [25]. One of the critical determinants found in our study is the need for feedback by the patients to the HCP for the HCP to stay personal.

### Reflection in Asynchronous eHealth Coaching

In the current study, the HCPs described how the asynchronous eHealth coaching provided both patients and HCPs with the chance to reflect during the time interval between questions and answers. The value of having time for reflection has not, to our knowledge, been evaluated in other eHealth studies. Most studies investigating asynchronous eHealth communication have examined how primary care providers can optimize their access to specialists [28]. An earlier systematic review looking at Web-based solutions compared asynchronous with synchronous eHealth consultations [29] Unfortunately, they only found a few studies of relevant quality, which meant that they could not make any conclusions about asynchronous versus synchronous solutions.

### Identifying Realistic Goals Based on Personal Barriers

Barriers to lifestyle changes can be difficult to detect. We speculate that these barriers change over time, and what seems realistic in an initial in-person meeting in a municipality setting might look different when reality strikes at home. This is an issue that might have a more significant impact on the lives of patients of low socioeconomic status [30]. We found that patients often perceive goal-setting as a process of creating long-term, distant and broader goals, which can give them a feeling of defeat if they fail. An essential part of the HCP’s job in eHealth is to assist patients in setting realistic, short-term, measurable subgoals to make sure distant goals do not decrease the patient’s self-efficacy. Self-efficacy is known to be a critical success factor for a lifestyle change. Apart from this, emphasizing the “small victories” is a key strategy used by many of the eHealth coaches, which makes good sense since self-efficacy can be increased by increasing a person’s feeling of control over the behavior. When these goals are appropriately structured, eHealth is unique in its ability to help individuals achieve measurable, realistic goals [31].

### Staying Connected in Asynchronous Coaching

Most of the HCPs revealed how important it was to be personal in their communications. Earlier studies have shown a high adherence to hybrid asynchronous collaborative eHealth coaching tools, which might be due to patients taking responsibility and feeling in charge [8,9]. In this study, when the relationship was inactive due to the patient not interacting, the HCPs found many ways to re-establish communication, such as being personal, reading the patient and using open questions. Being a trusted person involves providing nonjudgmental emotional support through conversation, reflective listening, touch, and physical presence when helping patients and relatives through difficult times [32]. Translating this into eHealth coaching is challenging, but personal nonjudgmental coaching seems essential in order not to lose patient feedback. One important takeaway message from the HCPs is that no matter how trivial a message or a video might seem, in order to make a difference for the patient it should always be personalized if possible. Personalization can be done by using information found in the message dialogue history. The need for personalization in asynchronous coaching is in line with other studies examining the delivering of standardized information [33]. The possibility of sending relevant and timely individual videos both from HCPs and patients might also be of vital importance. Using asynchronous video messaging might be of special importance in breaking down some of the obstacles presented by low health literacy. The question of how this would affect motivation and change behavior is still not scientifically documented.

### Strengths and Limitations of this Study

This is the first qualitative research study to analyze how HCPs, coaching via a hybrid, collaborative eHealth tool, perceive what is essential for successful lifestyle change among patients. The findings of this study are relevant and are expected to be of more general use in future research regarding the effectiveness and implementation challenges of collaborative eHealth solutions. However, in our study all HCPs were female, and even though saturation of central themes and subthemes was achieved in this group, more heterogeneity of eHealth coaches may lead to additional insights. Further dissemination of the eHealth tool used here along with other collaborative eHealth tools will also demand more research as they will not be applicable in all health care systems.

A limitation of this study is also the lack of methodological triangulation, as we only studied the perspective of the health care professionals and did not examine the patient perspective nor quantify the aspects revealed. For this reason, further studies using questionnaires and quantitative outcomes are suggested.

### Conclusion

Successful eHealth coaching requires establishing and maintaining an empathic relationship. HCPs found it of paramount importance to get to know the patient first, preferably in an initial face-to-face meeting and to provide both relational communication and goal-oriented coaching when using eHealth solutions. The asynchronous interaction aspect presented challenges because of the delay in response times (ie, both ways), but it also presented opportunities for reflection before answering. The future quality of the HCP-patient interaction will need attention if patients are to fully benefit from behavior change techniques made possible by eHealth coaching. Our findings suggest that it will be of great value to the future development of collaborative eHealth interventions if the quality of the HCP-patient interaction is taken further into account. This includes focusing on educating the health professionals about their empathic role as eHealth coaches and by strengthening their ability to communicate with empathy via new digital tools. This study emphasizes that collaborative eHealth tools used in empathic patient care can constitute an effective way to deliver health care services compassionately in the future for patients needing to implement lifestyle changes.

## Appendix

Multimedia Appendix 1 The Template for Intervention Description and the Replication checklist for the eHealth solution LIVA.

## Abbreviations

eHealth: electronic health
GP: general practitioner
HCP: health care professional
