# Supplementary material for: Determinants of Successful eHealth Coaching for Consumer Lifestyle Changes: Qualitative Interview Study Among Health Care Professionals
Source: J Med Internet Res. 2018 Jul 5;20(7):e237. doi: 10.2196/jmir.9791 (PMC6053604; doi:10.2196/jmir.9791)
Supplement: Multimedia Appendix 1 [file jmir_v20i7e237_app1.pdf]

Appendix 1. (TIDieR) checklist[a] for the e-health solution LIVA

TiDieR checklist item	Description
**What**<br><br>E-health coaching sessions	The healthcare professionals (HCP) had received training in setting SMART[b] goals with the patients using the collaborative e-health solution LIVA to set up action and coping plans that addressed barrier identification and problem solving. The training was led by a HCP/e-health coach with more than ten years' experience. Patients who wanted to improve their lifestyle received one or two face-to face meetings with the HCP, followed by asynchronous e-health coaching based on dialog by means of text or video. The e-health coaching sessions addressed the patient's data registrations, goal-setting and questions regarding: diet, exercise and lifestyle plans, and took chronic diseases into consideration. The LIVA app is set up with short explanations of different functions and notifications and reminders to the patients to register and give feedback on the health coaching. The sessions provide the user with tailored information in relation to their status, specific focus on goals and recommendations on how to improve their behaviours.<br><br>Included BCT[c] from CALO-RE taxonomy (hereafter referred to as BCT): provide information on consequences of the behaviour in *general* and *to the individual*, goal setting: behaviour and outcome, action planning, barrier identification/problem solving, set graded tasks, prompt review of behavioural goals, prompt review of outcome goals, prompt rewards contingent on effort or progress towards behaviour, prompting generalization of a target behaviour, provide feedback on performance.
Goals and inputs	The goals and inputs described underneath this column are available to the patient, who can choose his/her personal focus area, set specific concrete goals and keep record of specified behaviour(s) by reporting on them on a daily, weekly or monthly basis together with the HCP. This allows the user and the HCP to follow progress or setbacks as the numbers and registrations get visualized with graphs and curves (BCT: goal setting: behaviour and outcome, prompt self-monitoring of behaviour, provide feedback on performance, prompt practice).
Dietary goals and plans - input	Dietary goals and plans can include different scenarios, i.e. eating more healthy foods such as vegetables with the main courses, eating less carbs, fat and sugar or eating one biscuit instead of five for a snack.
Physical activity goals and plans - input	Realistic goals are set and recording of type and time for executing any given physical activity. The user receives advice and/or a video about activities in a variety of contexts to foster physical activity as a more integrated part of the person's life. (BCT: provide instruction on how to perform the behaviour, prompting generalization of a target behaviour, relapse prevention/coping planning).

Life goals - input	Goals for a healthy, joyful life as the patient sees it, i.e. daily life with less stress, stronger social bonds with friends and family, coping skills for diseases, etc.
Weight - input	Set current weight and goal for a lower/higher weight and register new measurements on a daily, weekly or monthly basis.
Steps - input	When downloading the app, the user can accept that their information on steps recorded on a smart phone is imported directly, and tailored messages about progress towards a set goal appear simultaneously (BCT: teach to use prompts/cues).
Pain, Sleep and Mood – input	Give daily or weekly feedback on pain, sleep and mood which can affect the ability to perform a given behaviour (BCT: relapse prevention/coping planning).
Smoking – input	Set goals to bring down number of cigarettes smoked on a daily basis, leading to cessation.
Blood glucose, cholesterol and lung capacity - input	Keeping a record of specified measures expected to be influenced by the different behaviour changes addressed. In LIVA this includes blood glucose, cholesterol and lung capacity. (BCT: prompt self-monitoring of behavioural outcome, provide information on consequences of the behaviour in *general* and *to the individual*).
Forum	Online forum where the users can exchange knowledge, gain social support and build new relationships, and the health coach can add advice to the forum users (BCT: plan social support/social change).
**Who provided**	HCP with basic training as nurses, physiotherapists, dieticians and occupational therapists were performing the health coaching.
**How**	Individually delivered via the App (iOS/Android) or web.
**Where**	Initial face-to-face meetings in the municipality health centres. Then solely e-health delivery.
**When and how much**	The initial face-to-face consultation(s) with a health coach and/or a nurse lasted approx. 45-60 minutes. The subsequent asynchronous e-health coaching sessions were carried out once a week in the first three months and then for maintenance every third week during the last nine months. In the year thereafter, the patient could receive two e-health coaching sessions and use LIVA as a personal behavioural change tool. (BCT: use of follow up prompts)
**Tailoring**	Every patient received personalized e-health coaching sessions from their designated health coach. The feedback given was based on the patient’s

	inputs on LIVA.
**Modifications**	During the study, the forum function on LIVA was changed so that the users could choose to join an online forum targeted at their specific location. One municipality health centre had just started to invite LIVA users to a 60 min. social group meeting in the health centre held every sixth week.

[a]TIDieR template for intervention description and replication [18]
[b]SMART: specific, measurable, agreed upon, realistic, and time-based goals.
[c]BCT: behaviour change technique.
[d]CALO-RE: Coventry, Aberdeen and London-Refined taxonomy [19]
